# Slitrk Missense Mutations Associated with Neuropsychiatric Disorders Distinctively Impair Slitrk Trafficking and Synapse Formation

**DOI:** 10.3389/fnmol.2016.00104

**Published:** 2016-10-20

**Authors:** Hyeyeon Kang, Kyung Ah Han, Seoung Youn Won, Ho Min Kim, Young-Ho Lee, Jaewon Ko, Ji Won Um

**Affiliations:** ^1^Department of Physiology and BK21 PLUS Project for Medical Science, Yonsei University College of MedicineSeoul, Korea; ^2^Department of Chemistry, Korea Advanced Institute of Science and TechnologyDaejeon, Korea; ^3^Graduate School of Medical Science and Engineering, Korea Advanced Institute of Science and TechnologyDaejeon, Korea; ^4^Department of Biochemistry, College of Life Science and Biotechnology, Yonsei UniversitySeoul, Korea

**Keywords:** Slitrks, schizophrenia, Tourette’s Syndrome, synapse formation, transmembrane protein

## Abstract

Slit- and Trk-like (Slitrks) are a six-member family of synapse organizers that control excitatory and inhibitory synapse formation by forming *trans*-synaptic adhesions with LAR receptor protein tyrosine phosphatases (PTPs). Intriguingly, genetic mutations of Slitrks have been associated with a multitude of neuropsychiatric disorders. However, nothing is known about the neuronal and synaptic consequences of these mutations. Here, we report the structural and functional effects on synapses of various rare *de novo* mutations identified in patients with schizophrenia or Tourette syndrome. A number of single amino acid substitutions in Slitrk1 (N400I or T418S) or Slitrk4 (V206I or I578V) reduced their surface expression levels. These substitutions impaired glycosylation of Slitrks expressed in HEK293T cells, caused retention of Slitrks in the endoplasmic reticulum and *cis*-Golgi compartment in COS-7 cells and neurons, and abolished Slitrk binding to PTPδ. Furthermore, these substitutions eliminated the synapse-inducing activity of Slitrks, abolishing their functional effects on synapse density in cultured neurons. Strikingly, a valine-to-methionine mutation in Slitrk2 (V89M) compromised synapse formation activity in cultured neuron, without affecting surface transport, expression, or synapse-inducing activity in coculture assays. Similar deleterious effects were observed upon introduction of the corresponding valine-to-methionine mutation into Slitrk1 (V85M), suggesting that this conserved valine residue plays a key role in maintaining the synaptic functions of Slitrks. Collectively, these data indicate that inactivation of distinct cellular mechanisms caused by specific Slitrk dysfunctions may underlie Slitrk-associated neuropsychiatric disorders in humans, and provide a robust cellular readout for the development of knowledge-based therapies.

## Introduction

Neuropsychiatric disorders such as schizophrenia, autism spectrum disorders (ASDs), and Tourette syndrome usually comprise heterogeneous and complex clinical syndromes with largely unknown etiologies (State, 2011; State and Levitt, 2011). Although epidemiological and descriptive studies have attempted to formulate etiological hypotheses to account for a subset of neuropsychiatric disorders, genetic studies have clearly demonstrated the high heritability of these disorders, albeit with complex inheritance pattern (Heinzen et al., 2015). To further complicate matters, genetic causation can range from a simple point mutation in a single gene to polygenetic causes that enlist an unknown mode of inheritance, incomplete penetrance, variable expressivity, epistasis, and/or etiological heterogeneity (Heinzen et al., 2015). Faced with this daunting challenge, researchers seeking to attain the conceptual advances necessary to design effective and precise therapeutics, critically require a more detailed comprehension of brain function (Insel and Landis, 2013). Rapid advances in human genome sequencing techniques have contributed significantly to the elucidation of candidate genes that are highly associated with a variety of neuropsychiatric disorders (Medland et al., 2014; Heinzen et al., 2015). In particular, large-scale, genome-wide association studies of patients suffering from various neuropsychiatric diseases have identified copy number variants, single-nucleotide polymorphisms and a variety of point mutations (State, 2011; State and Levitt, 2011; Heinzen et al., 2015). Intriguingly, a number of synaptic genes have frequently been identified as susceptibility factors, supporting the well-accepted ‘synaptopathy’ hypothesis, which posits that distinct synaptic dysfunctions constitute core features of various neuropsychiatric disorders (Brose et al., 2010). Not surprisingly, a host of synaptic adhesion molecules (e.g., neurexins and neuroligins) have been recognized as candidate contributors to various neuropsychiatric disorders, given their central importance in synaptic functions (Sudhof, 2008; Missler et al., 2012; Ko J. et al., 2015). However, how dysfunctions of most synaptic proteins lead to these devastating disorders remains poorly understood. In particular, why identical genetic factors are commonly found in clinically separable neuropsychiatric disorders, a finding that could account for the high comorbidity of a subset of brain disorders (Keezer and Sander, 2016; Keezer et al., 2016), has remained puzzling.

Slit- and Trk-like (Slitrk) proteins constitute a family of leucine-rich repeat (LRR)-containing synaptic adhesion molecules that are highly expressed in the central nervous system (Aruga and Mikoshiba, 2003; Aruga et al., 2003; Beaubien and Cloutier, 2009; de Wit et al., 2011; Ko, 2012). They possess a common structural architecture composed of tandem LRR domains (LRR1 and LRR2), a single transmembrane domain, and a divergent cytoplasmic region (Aruga and Mikoshiba, 2003). Functionally, they control excitatory and inhibitory synapse formation by interacting with LAR-RPTPs (leukocyte common antigen-related receptor protein tyrosine phosphatases, PTP) through the LRR1 domain (Takahashi et al., 2012; Um and Ko, 2013; Yim et al., 2013; Han et al., 2016a; see Um et al., 2014a, for complex structure). Slitrk3 specifically regulates inhibitory synapse development through binding to PTPδ, whereas other Slitrks organize excitatory synapse development through binding to PTPσ (Takahashi et al., 2012; Yim et al., 2013). Like other synaptic adhesion proteins, a subset of Slitrk point mutants have been linked to schizophrenia, ASDs, Tourette syndrome, or obsessive-compulsive disorder (OCD; reviewed in Ko, 2012; Um and Ko, 2013). For example, Slitrk1 has been postulated to be causative for Tourette syndrome (Abelson et al., 2005; Grados and Walkup, 2006), although subsequent studies (Keen-Kim and Freimer, 2006; Chou et al., 2007; Fabbrini et al., 2007; Scharf et al., 2008) have questioned the validity of conclusions reached by these earlier studies (Keen-Kim and Freimer, 2006; Chou et al., 2007; Fabbrini et al., 2007; Scharf et al., 2008). Moreover, several non-synonymous, rare variants of Slitrk1, Slitrk2, and Slitrk4 were recently reported to be associated with schizophrenia or OCD spectrum disorders (Zuchner et al., 2006; Piton et al., 2011; Ozomaro et al., 2013). However, it remains to be determined whether these mutations are functionally significant or represent benign polymorphisms.

Here, we systematically investigated the effects of four Slitrk1, five Slitrk2, and two Slitrk4 missense mutations on biochemical properties, surface transport, ligand-binding activity, and synaptogenic activities in cultured hippocampal neurons. We show that a subset of Slitrk1 (N400I and T418S) and Slitrk4 (V206I and I578V) mutations impair the biochemical and cell-biological properties of the corresponding wild-type (WT) Slitrks, through a common loss-of-function mechanism that basically traps them intracellularly, blocking their surface transport and ligand binding, and abolishing their synapse-promoting activity. By comparison, a Slitrk2 V89M mutation did not alter any of these parameters; instead it acted through a gain-of-function mechanism to compromise the ability of WT Slitrk2 to restore deficits in synapse density observed in Slitrk2-deficient neurons. Intriguingly, the analogous Slitrk1 point mutant (Slitrk1[V85M]) also displayed an impaired ability to rescue synaptogenic deficits in Slitrk1-deficient neurons. Our data reveal the underlying mechanisms by which a subset of Slitrk missense mutations observed in patients with neuropsychiatric disorders induce distinct gain- and loss-of-function phenotypes, and may provide a partial correlation with the clinical phenotypes shown in Slitrk dysfunction-associated disorders.

## Materials and Methods

### *In silico* Analysis

Previously identified Slitrk missense mutations implicated in neuropsychiatric disorders were analyzed using four different prediction programs. (1) *PolyPhen-2*, available via the Web server^1^, predicts the functional significance of an allele replacement from its individual features employing a Naïve Bayes classifier, trained using supervised machine learning. Mutations whose posterior probability scores are associated with estimated false-positive rates (FPR) at or below the first (lower) FPR are predicted to be ‘probably damaging’. Mutations with posterior probabilities associated with FPR at or below the second (higher) FPR are predicted to be ‘possibly damaging’. Mutations with an estimated FPR above the second (higher) FPR value are classified as ‘benign’. (2) *PROVEAN (Protein Variation Effect Analyzer)*, available via the Web server^2^, clusters BLAST hits using its CD-HIT module based on a global sequence identity parameter of 75%. The supporting sequence set, representing the top 30 clusters of closely related sequences, is used to generate predictions. For each supporting sequence, a delta alignment score is computed and used to determine the PROVEAN score. If the PROVEAN score is less than or equal to -2.5 (predefined threshold), the protein variant is considered ‘deleterious’; variants with scores greater than -2.5 are considered ‘neutral’. (3) *MutationAssessor* was automatically run using the Web server^3^, as previously described (Reva et al., 2011). (4) *PANTHER (Protein Analysis Through Evolutionary Relationships)*, available through the Web server^4^, generates SubPSEC scores, which are continuous values from 0 (neutral) to about -10 (most likely deleterious). A P_deleterious_ value of 0.5 corresponds to a SubPSEC score of -3. The probability that a given variant will cause a deleterious effect on protein function is estimated from P_deleterious_ such that a SubPSEC score of -3 corresponds to a P_deleterious_ of 0.5 (Mi et al., 2016). ‘n.d.’ in PANTHER indicates that the position does not align to Hidden Markov Model (HMM) libraries.

### Structural Modeling

Structures of human Slitrk2 (LRR1, aa D27–P264; and LRR2, aa S342–P579) and human Slitrk4 (LRR1, aa N28–P64; and LRR2, aa P343–P581) were modeled through the SWISS-MODEL server using human Sltirk1 LRR1 (PDB ID: 4RCA), mouse Slitrk2 LRR1 (PDB ID: 4Y61), and human Slitrk1 LRR2 (PDB ID: 4RCW) as templates. All drawings depicting molecular structures were prepared using PyMOL (PyMOL Molecular Graphics System) (Um et al., 2014a).

### Construction of Expression Vectors

pDisplay-Slitrk1 mutants (V85M, N400I, T418S, R584K, S593G), pDisplay-Slitrk2 mutants (R32L, V89M, S549F, S601P, L626F), and pDisplay-Slitrk4 mutants (V206I, I578V) were generated with a site-directed mutagenesis kit (Stratagene) using the corresponding WT pDisplay constructs as templates. The following constructs were previously described: pDisplay-Slitrk1 WT, pDisplay-Slitrk2 WT, pDisplay-Slitrk4 WT, L-315 sh-Slitrk1, L-315 sh-Slitrk2, and L-315 sh-Slitrk4 (Yim et al., 2013); and pVL1393-PTPδ (Um et al., 2014a). The BFP-KDEL vector was purchased from Addgene (construct #49150).

### Antibodies

The following antibodies were obtained from the indicated commercial sources: mouse monoclonal anti-HA (clone HA-7; Covance), rabbit polyclonal anti-HA (H6908; Sigma), mouse monoclonal anti-α-tubulin (clone DM1A; Sigma), rabbit polyclonal anti-actin (A2066; Sigma), goat polyclonal anti-EGFP (Rockland), and mouse monoclonal anti-GM130 (clone 35/GM130; BD Transduction Laboratories). The anti-synapsin antibody was previously described (Han et al., 2016b).

### Heterologous Synapse-Formation Assay

Heterologous synapse-formation assays were performed using HEK293T cells as previously described (Ko et al., 2009; Um et al., 2014b). In brief, HEK293T cells were cotransfected with EGFP or HA-Slitrk1 WT, HA-Slitrk1 point mutants, HA-Slitrk2 WT, HA-Slitrk2 point mutants, HA-Slitrk4 WT, or HA-Slitrk4 point mutants (described in ‘Construction of expression vectors’) using the FuGene reagent (Roche). After 48 h, transfected HEK293T cells were trypsinized and seeded onto DIV9 hippocampal neuron cultures. Cells were further cocultured for 72 h and then double-immunostained with anti-HA and anti-synapsin antibodies at DIV12 as described previously (Ko J.S. et al., 2015). All images were acquired with a confocal microscope. For quantifications, the contours of transfected HEK293T cells were chosen as the region of interest. The fluorescence intensity of synapsin puncta normalized to the area of each HEK293T cell was quantified for both red and green channels using MetaMorph Software (Molecular Devices).

### Glycosylation Assay

Solubilized proteins from HEK293T cells transfected with expression plasmids for WT or mutant Slitrks, were first denatured by adding 10x denaturing buffer (New England Biolabs) and heating to 100°C for 10 min. For endoglycosidase H (Endo H) treatment, denatured protein was treated with 1 μl of enzyme and incubated at 37°C for 1 h. For PNGase F treatment, denatured protein was incubated with 1 μl of enzyme in the presence of 2 μl of NP-40 (100%) at 37°C for 1 h. Thereafter, enzyme-treated proteins, together with an equal amount of untreated proteins, were analyzed by immunoblotting with the indicated antibodies followed by enhanced chemiluminescence (ECL) detection.

### Biotinylation Assay

Biotinylation experiments in HEK293T cells were performed as previously described (Han et al., 2016b). Briefly, HEK293T cells transfected with the indicated Slitrk plasmids were washed three times with ice-cold PBS containing 1 mM MgCl_2_ and 1 mM CaCl_2_. Sulfo-NHS-LC-biotin (1 mg/ml) was then added, and cells were kept at 4°C for 30 min. After incubation, cells were washed three times with PBS plus 100 mM glycine to quench and remove excess biotin. Purified membrane proteins were then incubated with Neutravidin (Thermo Scientific) overnight at 4°C. After three washes with PBS, proteins were eluted with sample buffer and analyzed by immunoblotting.

### Primary Neuronal Culture, Transfection, and Immunocytochemistry

Rat hippocampal cultures were prepared from embryonic day 18 (E18) embryos as described previously (Ko et al., 2011; Um et al., 2016). All experimental protocols using pregnant rats were approved by the Institutional Animal Care and Use Committee of Yonsei University College of Medicine. For overexpression, hippocampal neurons were transfected with various Slitrks vector using CalPhos Kit (Clontech) at DIV10 and immunostained at DIV14. For knockdown of Slitrk1, Slitrk2, or Slitrk4, hippocampal neurons were co-transfected with the corresponding L-315 sh-Slitrk vector at DIV8 and immunostained at DIV14, as indicated in the figure legends. For immunocytochemistry, cultured neurons were first fixed with 4% paraformaldehyde/4% sucrose for 10 minutes at room temperature and then permeabilized with 0.2% Triton X-100 in PBS for 5 min at 4°C. Fixed, permeabilized neurons were then blocked by incubating with 3% horse serum/0.1% crystalline grade bovine serum albumin (BSA) in PBS for 30 min at room temperature, and incubated with the indicated primary and secondary antibodies in blocking solution for 1 h each at room temperature.

### Neurite Outgrowth Assay

Hippocampal neurons prepared from E18 rat embryo were transfected with WT Slitrk, Slitrk[N400I] or Slitrk[T418S] together with pEGFP-N1 vector at DIV3 and immunostained with anti-EGFP antibodies at DIV6. Fluorescent images of neurons were randomly captured and neurite length was analyzed using MetaMorph software (Molecular Devices).

### Confocal Microscopy Image Acquisition and Analysis

Transfected neurons were randomly chosen and acquired at constant imaging settings using a confocal microscope (LSM700; Carl Zeiss) with a 63× objective lens. Z-stack images obtained at 0.1μm intervals by confocal microscopy were converted to maximal projections, and the size and density of presynaptic terminals were analyzed using MetaMorph software. All images were separated into different color channels (red and green), and red-colored images were transformed into an image in grayscale mode using Photoshop (Adobe). After selecting one or two primary dendrites from neurons in a single image frame, dendrite lengths were recorded and dendritic regions of interest were manually traced in MetaMorph software and saved for puncta measurements (in rgn file format). A constant intensity threshold that excluded diffuse nonsynaptic signals but included synaptic signals (90; range, 0–255) was applied to all gray images. The saved dendritic regions were loaded, calibrated, and measured using the ‘integrated morphometry analysis’ option. The linear density of synapsin clusters was determined from calculated total puncta numbers, normalized to 10 μm length of dendrite. For puncta size and intensity measurements, normalized puncta areas and averaged puncta intensities were calculated and exported automatically to the Excel program (Microsoft). All quantitative analyses were performed in a blinded manner.

### Statistics

All data are expressed as means ± SEM. All statistical analyses were performed using SPSS Statistics 23 (IBM, Armonk, NY, USA). The normal distribution of the data was investigated by *p*-values from Kolmogorov-Smirnov test (obtained *p*-values > 0.05, except the data in **Figures 6C** and **10B**). Thus, these data were statistically evaluated using one-way analysis of variance (ANOVA), using cell numbers (>10) or the number of experiments (>3) as the basis for ‘*n*’. For the **Figures 6C** and **10B**, the data were statistically assessed using a non-parametric Kruskal-Wallis test.

## Results

**FIGURE 1:**
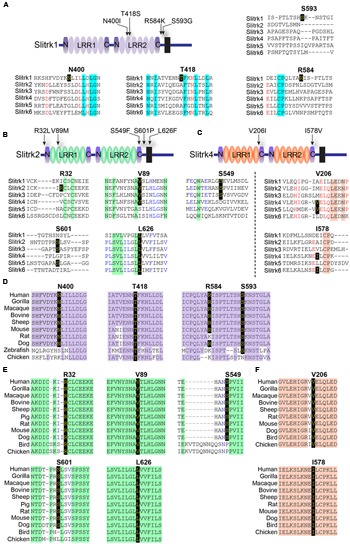
**Alignment and conservation across different species of Slitrk1, Slitrk2, and Slitrk4 (Slit- and Trk-like) residues that are mutated in human patients with schizophrenia, Tourette syndrome, or trichotillomania. (A–C)** Alignment of human Slitrk amino acids surrounding the mutated residues found in human patients with neuropsychiatric disorders. The target mutated resides are indicated in bold. Schematic drawings of the entire domain organization of human Slitrk1 **(A)**, Slitrk2 **(B)**, and Slitrk4 **(C)** are shown. **(D-F)** Similarity or identity of mutated residues investigated in the current study was determined by analyzing the amino acid sequences of human Slitrk1 **(D)**, Slitrk2 **(E)**, and Slitrk4 **(F)** deposited in the National Center for Biotechnology Information (NCBI) database. Identical residues across various species are indicated in yellow letters on a black background. The following GenBank accession numbers were utilized for sequence alignment: Slitrk1/human, NP_443142; Slitrk1/gorilla, XP_004054683; Slitrk1/macaque, NP_001247716; Slitrk1/bovine, XP_002691955; Slitrk1/sheep, XP_004012247; Slitrk1/mouse, EDL00537; Slitrk1/rat, NP_001100753; Slitrk1/dog, XP_542628; Slitrk1/zebrafish, XP_687093; Slitrk1/chicken, XP_416993; Slitrk2/human, NP_001137482; Slitrk2/gorilla, XP_004065021; Slitrk2/macaque, NP_001248149; Slitrk2/bovine, XP_015325702; Slitrk2/sheep, XP_004022343; Slitrk2/pig, XP_013841934; Slitrk2/rat, NP_001101057; Slitrk2/mouse, AAI12407; Slitrk2/dog, XP_013967295; Slitrk2/bird, XP_012428589; Slitrk2/chicken, XP_420364; Slitrk4/human, NP_001171679; Slitrk4/gorilla, XP_004065019; Slitrk4/macaque, XP_001086308; Slitrk4/bovine, XP_005227618; Slitrk4/sheep, XP_004022346; Slitrk4/rat, NP_001100417; Slitrk4/mouse, AAI17892; Slitrk4/dog, XP_005641950; Slitrk4/bird, XP_012428616; and Slitrk4/chicken, XP_015134020.

A variety of rare Slitrk point mutations have been found to be associated with schizophrenia or OCD spectrum disorders (Proenca et al., 2011; Ko, 2012; Um and Ko, 2013). Because one of the aims of this study was to evaluate the functional consequences of *SLITRK* gene mutations for neuropsychiatric disorders, we focused on only non-synonymous, missense mutations in this study. Eleven missense mutations have previously been reported to be linked to neuropsychiatric disorders (Zuchner et al., 2006; Piton et al., 2011; Ozomaro et al., 2013). These include N400I, T418S, R584K, and S593G in human Slitrk1; R32L, V89M, S549F, S601P, and L626F in human Slitrk2; and V206I and I578V in human Slitrk4 (**Figures 1A–C**). The L626F mutation in human Slitrk2 also exists in other human Slitrks at equivalent positions (**Figure 1B**), but none of the other residues exhibit complete sequence identity across the six Slitrk members (**Figures 1A–C**). Notably, all four mutated residues identified in human Slitrk1 are unique to Slitrk1 (**Figure 1A**). However, most of these residues are quite evolutionarily conserved among various species, implying their possible functional significance (**Figures 1D–F**). To draw inferences regarding the structural and functional importance of these single amino acid substitutions, we employed the widely used PolyPhen2 (Kumar et al., 2009), PANTHER (Thomas and Kejariwal, 2004), SIFT (Adzhubei et al., 2010), and MutationAssessor (Reva et al., 2011) software packages (**Table 1**). Interestingly, none of the Slitrk missense mutations that are the focus of this study were consistently predicted to be either benign or have deleterious impacts on the stability and function of human Slitrks by the four different *in silico* prediction tools (**Table 1**).

**Table 1:** Prediction of functional effect of Slit- and Trk-like (Slitrk) mutations using four different bioinformatics tools.

Gene	amino acid change	Cohort	PolyPhen-2	PROVEAN	Mutation Assessor	PANTHER		Reference
						SubPSEC Score	P_deleterious_	
Slitrk1	V85M	n.a.	Possibly damaging (0.855)	Neutral (-0.968)	Medium (2.31)	n.d.	n.d.	This study
Slitrk1	N400I	TS	Benign (0.162)	Deleterious (-2.943)	Low (1.93)	-2.40948	0.35652	Ozomaro et al., 2013
Slitrk1	T418S	TS	Benign (0.115)	Neutral (-0.274)	Neutral (0.505)	n.d.	n.d.	Ozomaro et al., 2013
Slitrk1	R584K	TTM	Benign (0)	Neutral (0.220)	Neutral (-0.485)	-0.25178	0.06019	Zuchner et al., 2006
Slitrk1	S593G	TTM	Benign (0.014)	Neutral (-1.596)	Medium (2.28)	-1.94135	0.25757	Zuchner et al., 2006
Slitrk2	R32L	SCZ	Benign (0.035)	Neutral (0.487)	Neutral (0.415)	n.d.	n.d.	Piton et al., 2011
Slitrk2	V89M	SCZ	Probably damaging (0.984)	Neutral (-0.932)	Low (1.545)	-2.59958	0.40121	Piton et al., 2011
Slitrk2	S549F	SCZ	Benign (0.203)	Deleterious (-2.957)	Low (0.985)	-3.12094	0.5302	Piton et al., 2011
Slitrk2	S601P	ASD	Benign (0)	Neutral (-0.205)	Neutral (0.5)	-1.47548	0.1788	Piton et al., 2011
Slitrk2	L626F	SCZ	Probably damaging (1)	Deleterious (-2.780)	Medium (1.975)	n.d.	n.d.	Piton et al., 2011
Slitrk4	V206I	SCZ	Probably damaging (0.998)	Neutral (-0.404)	Neutral (0.6)	n.d.	n.d.	Piton et al., 2011
Slitrk4	I578V	SCZ	Benign (0.012)	Neutral (0.173)	Neutral (0.425)	n.d.	n.d.	Piton et al., 2011

*ASD, Autism spectrum disorder; n.a., not applicable; n.d., not determined; PANTHER, Protein Analysis Through Evolutionary Relationships; PolyPhen-2, Polymorphism Phenotyping v2; PROVEAN, Protein Variation Effect Analyzer; SCZ, Schizophrenia; SIFT, Sorting Intolerant From Tolerant; TS, Tourette’s syndrome; TTM, Trichotillomania.*

### Prediction of Structural Phenotypes Produced by Slitrk Missense Mutations, As Reflected in Protein Folding and Three-Dimensional (3D) Structure

**FIGURE 2:**
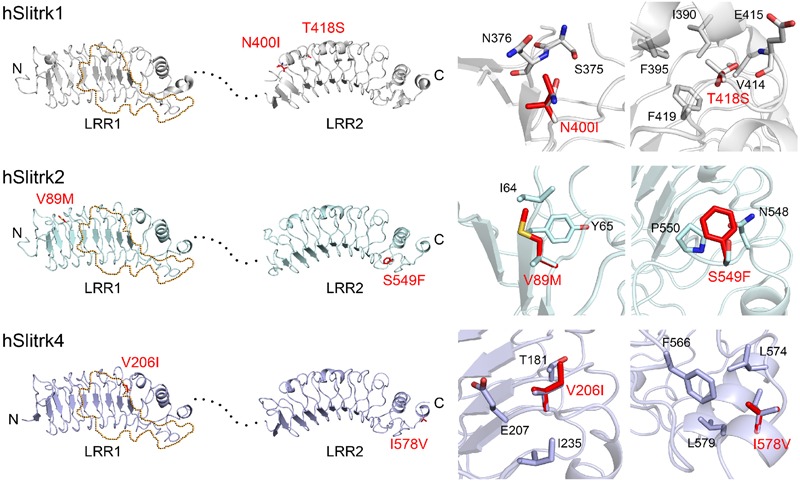
**Structural modeling of Slitrk1, Slitrk2, and Slitrk4 residues that are mutated in human patients.** Overall structures of LRR1 and LRR2 domains of human Slitrk1, Slitrk2 and Slitrk4 (left). The crystal structure of human Slitrk1 LRR1/human PTPδ Ig1-3 complex (PDB ID: 4RCA) and human Slitrk1 LRR2 (PDB ID: 4RCW) were used for presenting the structures of human Slitrk1 LRR1 and LRR2. The structure of human Slitrk2 and Slitrk4 were modeled with SWISS-MODEL server. The residues corresponding to patients’ mutations described in the current study are presented as red sticks in the cartoon presentation. Human Slitrk1, Slitrk2, and Slitrk4 are colored gray, cyan and purple, respectively. Yellow dotted regions in the LRR1 domains of Slitrks indicate the LAR-RPTP binding surfaces based on the 3D structure of the human Slitrk1 LRR1/PTPδ Ig1-3 complex (PDB ID: 4RCA), and black dotted lines represent flexible linkers between LRR1 and LRR2 domains (left). Close-up views of mutated and neighboring residues in LRR domains (right).

Notably, two Slitrk1 mutations (N400I and T418S) are located in the LRR2 domain (**Figure 1A**). Crystal structure of human Slitrk1 LRR2 indicated that the residue N400 of human Slitrk1 forms a weak hydrogen bond with an amino group of the main chain of S375 and a hydroxyl group of the side chain of N376 on the neighboring loop (**Figure 2**). Therefore, mutating N400 to a nonpolar isoleucine (Ile) residue is likely to disrupt these interactions, possibly causing misfolding and aberrant protein trafficking (see below). The side chain of T418 in human Slitrk1 forms a hydrogen bond with a carboxyl group of the main chain of E415 and is involved in hydrophobic interactions with I390, F395, and F419 (**Figure 2**). Thus, a point mutation of T418 to serine (T418S) is also expected to disrupt these hydrophobic interactions. The other Slitrk1 mutations (R584K and S593G) and Slitrk2 mutations (S601P and L626F) are located outside major structural domains, consistent with the results of *in silico* analyses (**Figure 1A**; **Table 1**). R32L in human Slitrk2 is located immediately preceding the LRR1 domain (i.e., the terminal residue of the signal peptide; **Figure 1A**). Point mutations in human Slitrk2 (V89M or S549F) and human Slitrk4 (V206I or I578V) were predicted to have little effect on the 3D structures of individual Slitrks (**Figure 2**) (Um et al., 2014a).

### Biochemical and Ligand-Binding Phenotypes of Disease-Associated Slitrk Missense Mutants

**FIGURE 3:**
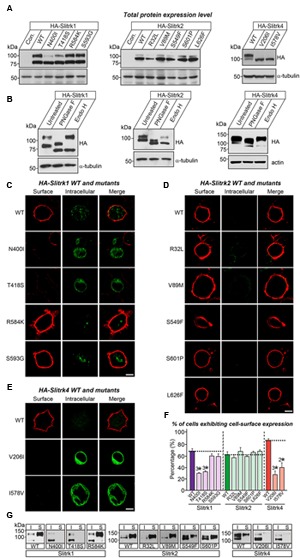
**Impaired glycosylation and surface trafficking of a subset of Slitrk mutations. (A)** Representative immunoblot images from HEK293T cells transfected with the indicated WT or mutant forms of HA-tagged Slitrk1, Slitrk2, or Slitrk4. Samples containing equal amounts of protein were resolved by SDS-PAGE and immunoblotted using anti-HA antibodies; α-tubulin was used for normalization. Molecular mass markers are labeled in kilodaltons. **(B)** Immunoblot analysis of Endo-H and PNGase F enzyme-digested lysates of HEK293T cells expressing WT HA-tagged Slitrk; α-tubulin or actin was used for normalization. Molecular mass markers are labeled in kilodaltons. **(C-E)** Surface expression analysis of HEK293T cells expressing WT or point mutant forms of HA-tagged Slitrk1 **(C)**, Slitrk2 **(D)**, or Slitrk4 **(E)**. Transfected cells were immunostained with mouse anti-HA antibodies (red) and detected with Cy3-conjugated anti-mouse secondary antibodies under non-permeabilized conditions, followed by permeabilization of cells. Cells were then stained first with rabbit anti-HA antibodies (green) and then with FITC-conjugated anti-rabbit secondary antibodies. Scale bar, 10 μm (applies to all images). **(F)** Quantification of the proportion of cells exhibiting surface expression of Slitrks. All data are shown as means ± SEMs (^2∗^*p* < 0.01; ^3∗^*p* < 0.001; ANOVA with *post hoc* Tukey’s test; *p*-value of WT Slitrk1 vs Slitrk1[N400I] = 0.000033; *p*-value of WT Slitrk1 vs Slitrk1[T418S] = 0.000065; *p*-value of WT Slitrk1 vs. Slitrk1[R584K] = 0.259; *p*-value of WT Slitrk1 vs. Slitrk1[S593G] = 0.34; *p*-value of WT Slitrk2 vs. Slitrk2[R32L] = 0.941; *p*-value of WT Slitrk2 vs. Slitrk2[V89M] = 0.948; *p*-value of WT Slitrk2 vs. Slitrk2[S549F] = 0.988; *p*-value of WT Slitrk2 vs. Slitrk2[S601P] = 0.997; *p*-value of WT Slitrk2 vs. Slitrk2[L626F] = 0.996; *p*-value of WT Slitrk4 vs. Slitrk4[V206I] = 0.000677; *p*-value of WT Slitrk4 vs. Slitrk4[I578V] = 0.003946). The numbers of cells counted (*n*) were as follows: WT Slitrk1, *n* = 360; Slitrk1[N400I], *n* = 327; Slitrk1[T418S], *n* = 412; Slitrk1[R584K], *n* = 496; Slitrk1[S593G], *n* = 333; WT Slitrk2, *n* = 345; Slitrk2[R32L], *n* = 321; Slitrk2[V89M], *n* = 371; Slitrk2[S549F], *n* = 360; Slitrk2[S601P], *n* = 313; Slitrk2[L626F], *n* = 319; WT Slitrk4, *n* = 256; Slitrk4[V206I], *n* = 181; and Slitrk4[I578V], *n* = 161. **(G)** Slitrk surface exposure on transfected HEK293T cells, analyzed by immunoblotting of affinity-purified, surface-biotinylated Slitrk proteins. Biotinylated cell surface proteins (S) and total lysate proteins (I) were assessed by immunoblot with anti-HA antibodies. Input, 5% of total lysates used in biotinylation experiments. Note that mutants of Slitrk1 (N400I and T418S) and Slitrk4 (V206I and I578V) exhibit impaired surface expression, as similarly observed in **(C-F)**.

**FIGURE 4:**
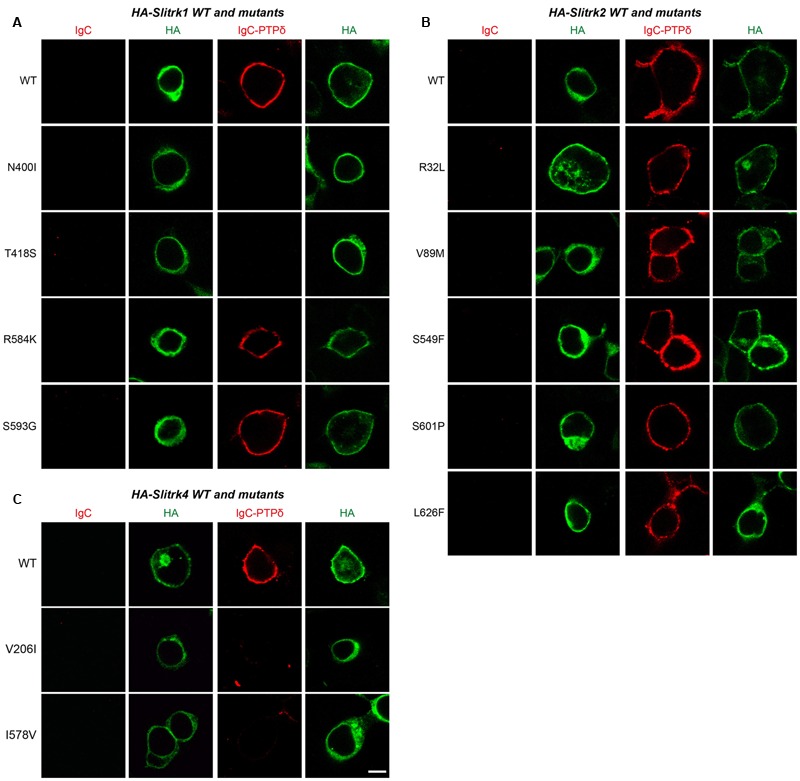
**Characterization of LAR-RPTP–binding properties of Slitrk mutations.** Representative images of cell-surface–binding assays. HEK293T cells expressing WT or mutant forms of HA-tagged Slitrk1 **(A)**, Slitrk2 **(B)**, or Slitrk4 **(C)** were incubated with 10 μg/ml control IgC or Ig-PTPδ, and then analyzed by immunofluorescence imaging of Ig-fusion proteins (red) and HA antibodies (green). Percentages of the cells exhibiting the binding of Ig-PTPδ were as follows: WT Slitrk1, 66.7%; Slitrk1[N400I], 32%; Slitrk1[T418S], 41%; Slitrk1[R584K], 62%; Slitrk1[S593G], 68.7%; WT Slitrk2, 71%; Slitrk2[R32L], 82%; Slitrk2[V89M], 81%; Slitrk2[S549F], 68.8%; Slitrk2[S601P], 80%; Slitrk2[L626F], 76%; WT Slitrk4, 81%; Slitrk4[V206I], 24%; and Slitrk4[I578V], 22.2%. Note that a subset of Slitrk mutants (Slitrk1[N400I], Slitrk1[T418S], Slitrk4[V206I] and Slitrk4[I578V]) exhibit reduced binding to Ig-PTPδ, paralleling their impaired surface transport (see **Figure 3**). Scale bar, 10 μm (applies to all images).

We next investigated the expression levels and intracellular trafficking of Slitrk mutants in non-neuronal cells (**Figure 3**). As is typically observed for numerous glycoproteins (Yim et al., 2013), immunoblot analyses of HEK293T cells transfected with expression vectors for HA-tagged, full-length Slitrks showed that WT Slitrk proteins were detectable as two discrete bands: approximately 75–100 kDa for Slitrk1 and Slitrk2, and 100–110 kDa for Slitrk4 (**Figure 3A**). Total protein expression levels of Slitrk point mutants were comparable to those of the corresponding WT Slitrks (**Figure 3A**). Strikingly, the mature protein levels of a subset of Slitrk1 mutants (N400I and T418S) and Slitrk4 mutants (V206I and I578V) were significantly decreased (**Figure 3A**). The upper band observed in lysates of Slitrk-expressing HEK293T cells represents fully glycosylated mature protein species that are resistant to Endo-H (which cleaves only immature sugars attached in the endoplasmic reticulum, ER) and are presumably presented to the cell surface, whereas the lower band represents glycosylated immature protein species that are sensitive to Endo-H and are undergoing processing in the ER compartment (**Figure 3B**). Immunoblot analyses of WT Slitrks treated with PNGase F, which removes all attached N-linked oligosaccharides, showed a band shift (**Figure 3B**), consistent with a previous report that Slitrks are highly glycosylated (Yim et al., 2013). We next examined the surface and intracellular protein levels of individual WT Slitrks and Slitrk point mutants in HEK293T cells (**Figures 3C–E**). In line with the biochemical data, two Slitrk1 mutants (N400I and T418S) and two Slitrk4 mutants (V206I and I578V), but not the other Slitrk mutants, displayed significantly reduced surface expression levels with complete trapping in intracellular compartment (**Figures 3C–F**). To reaffirm findings of immunofluorescence analyses in HEK293T cells, we performed biotinylation experiments. We found that the same mutations in Slitrk1 (N400I and T418S) and Slitrk4 (V206I and I578V) impaired the surface expression of the corresponding protein (**Figure 3G**). We then performed binding assays between recombinant Ig-fusion proteins of PTPδ (IgC-PTPδ) and HEK293T cells expressing HA-tagged Slitrks (**Figure 4**). Recombinant IgC-PTPδ robustly bound to HEK293T cells expressing various Slitrk mutants, except those expressing the Slitrk1/4 mutants with impaired surface expression (**Figures 4** and **3C–G**). These data suggest that a subset of Slitrk point mutations observed in neuropsychiatric patients causes improper biochemical processing and abnormal cellular trafficking in non-neuronal cells.

### Cellular Phenotypes of Disease-Associated Slitrk Missense Mutants

**FIGURE 5:**
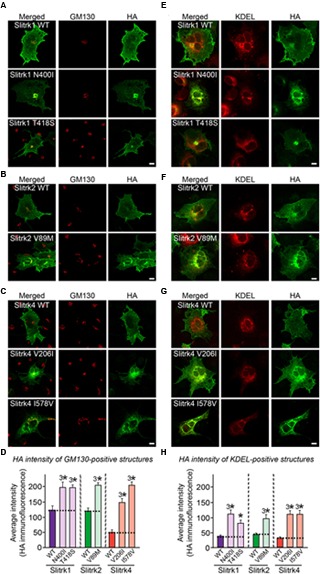
**Retention of Slitrk mutant proteins in the *cis*-Golgi and ER in COS-7 cells. (A-C)** Immunofluorescence staining of COS-7 cells demonstrates *cis*-Golgi retention of a subset of mutants. COS-7 cells were transfected with WT or mutant forms of HA-tagged Slitrk1 **(A)**, Slitrk2 **(B)**, or Slitrk4 **(C)**, and then stained with anti-HA (green) and anti-GM130 (red) antibodies. Scale bar, 10 μm (applies to all images). **(D)** Quantification of the average intensity of GM130-positive Slitrks. All data are shown as means ± SEMs (^3∗^*p* < 0.001; ANOVA with *post hoc* Tukey’s test; *p*-value of WT Slitrk1 vs Slitrk1[N400I] = 0.00053; *p*-value of WT Slitrk1 vs. Slitrk1[T418S] = 0.000383; *p*-value of WT Slitrk2 vs. Slitrk2[V89M] = 1.83943E-7; *p*-value of WT Slitrk4 vs. Slitrk4[V206I] = 8.0637E-9; *p*-value of WT Slitrk4 vs. Slitrk4[I578V] = 5.1018E-9). The numbers of cells counted were as follows: WT Slitrk1, *n* = 26; Slitrk1[N400I], *n* = 22; Slitrk1[T418S], *n* = 24; WT Slitrk2, *n* = 24; Slitrk2[V89M], *n* = 27; WT Slitrk4, *n* = 19; Slitrk4[V206I], *n* = 22; and Slitrk4[I578V], *n* = 23. **(E-G)** Immunofluorescence staining of COS-7 cells demonstrates ER retention of mutants. COS-7 cells were transfected with WT or mutant forms of HA-tagged Slitrk1 **(E)**, Slitrk2 **(F)**, or Slitrk4 **(G)**, together with BFP-KDEL (red; pseudo-colored). The cells were then stained with anti-HA (green). Scale bar, 10 μm (applies to all images). **(H)** Quantification of the average intensity of KDEL-positive Slitrks. All data are shown as means ± SEMs (^∗^*p* < 0.05, ^3∗^*p* < 0.001; ANOVA with *post hoc* Tukey’s test; *p*-value of WT Slitrk1 vs. Slitrk1[N400I] = 0.000093; p-value of WT Slitrk1 vs. Slitrk1[T418S] = 0.036; *p*-value of WT Slitrk2 vs. Slitrk2[V89M] = 0.000712; *p*-value of WT Slitrk4 vs. Slitrk4[V206I] = 0.000257; *p*-value of WT Slitrk4 vs. Slitrk4[I578V] = 0.000458). The numbers of cells counted were as follows: WT Slitrk1, *n* = 26; Slitrk1[N400I], *n* = 24; Slitrk1[T418S], *n* = 22; WT Slitrk2, *n* = 17; Slitrk2[V89M], *n* = 23; WT Slitrk4, *n* = 22; Slitrk4[V206I], *n* = 22; and Slitrk4[I578V], *n* = 21.

**FIGURE 6:**
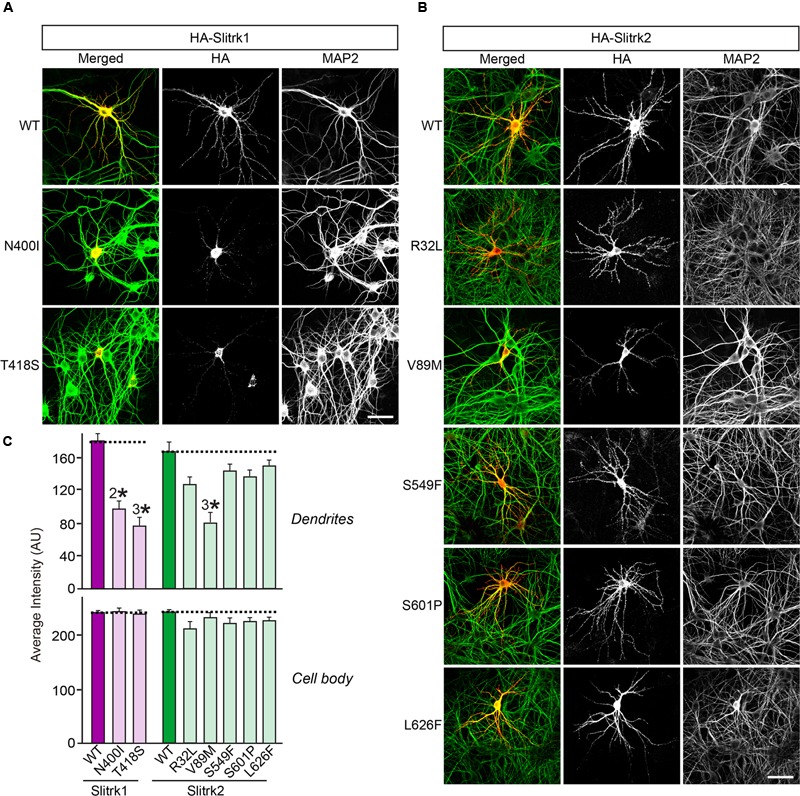
**Impaired dendritic targeting of Slitrk1[N400I], Slitrk1[T418S] and Slitrk2[V89M] mutants in cultured hippocampal neurons. (A-B)** Fluorescence images of hippocampal neurons transfected with wild-type (WT) HA-Slitrk1, HA-Slitrk1[N400I], HA-Slitrk1[T418S], WT HA-Slitrk2, HA-Slitrk2[R32L], HA-Slitrk2[V89M], HA-Slitrk2[S549F], HA-Slitrk2[S601P] or HA-Slitrk2[L626F] at DIV10. The transfected neurons at DIV14 were double-immunostained for antibodies against the somatodendritic marker MAP2 (green) and HA (red). Note that Slitrk1[N400I] and Slitrk1[T418S] are mainly observed in the periphery of soma regions of the transfected neurons, but not in dendritic regions. Meanwhile, Slitrk2[V89M] exhibits decreased dendritic targeting. Scale bar, 50 μm (applies to all images). **(C)** Dendritic targeting of WT HA-Slitrk1, HA-Slitrk1[N400I], HA-Slitrk1[T418S], WT HA-Slitrk2, HA-Slitrk2[R32L], HA-Slitrk2[V89M], HA-Slitrk2[S549F], HA-Slitrk2[S601P], or HA-Slitrk2[L626F] in hippocampal neurons was quantified by measuring average intensity of HA immunofluorescence in primary dendrites. The average intensity of Slitrk WT and mutants in soma region of the transfected neurons was also quantified. All data are shown as means ± SEMs (^2∗^*p* < 0.01; ^3∗^*p* < 0.001; a non-parametric Kruskal-Wallis test; *p*-value of WT Slitrk1 vs. Slitrk1[N400I] = 0.001; *p*-value of WT Slitrk1 vs. Slitrk1[T418S] < 0.001; *p*-value of WT Slitrk2 vs. Slitrk2[R32L] = 0.052; *p*-value of WT Slitrk2 vs. Slitrk2[V89M] = 0.000; *p*-value of WT Slitrk2 vs. Slitrk2[S549F] = 0.178; *p*-value of WT Slitrk2 vs. Slitrk2[S601P] = 0.169; *p*-value of WT Slitrk2 vs. Slitrk2[L626F] = 0.284). ‘*n*’ denotes the number of neurons as follows: WT Slitrk1, *n* = 16; Slitrk1[N400I], *n* = 19; Slitrk1[T418S], *n* = 16; WT Slitrk2, *n* = 16; Slitrk2[R32L], *n* = 14; Slitrk2[V89M], *n* = 14; Slitrk2[S549F], *n* = 13; Slitrk2[S601P], *n* = 14; and Slitrk2[L626F], *n* = 13.

The immunocytochemical results clearly indicate that a subset of Slitrk point mutants exhibits abnormal trafficking and decreased surface expression (**Figure 3**). To independently corroborate this interpretation, we analyzed the subcellular distribution of Slitrk mutants using immunofluorescence microscopy (**Figure 5**). First, we transfected COS-7 cells with WT or mutant Slitrks, and stained them with antibodies against HA and GM130 (a *cis*-Golgi marker) (**Figures 5A–C**). Consistent with the biochemical data, a subset of Slitrk mutant proteins showed distinct patterns of colocalization with GM130 (**Figures 5A–C**). The average fluorescence intensity of GM130-positive Slitrk mutant proteins was significantly increased than that of WT (**Figure 5D**). We also cotransfected COS-7 cells with WT and mutant Slitrks, together with a blue fluorescent protein-fused KDEL construct to visualize the ER compartment (**Figures 5E–G**). Again, we found that Slitrk mutant proteins showed overlapping colocalization with BFP-KDEL (**Figure 5H**). These data indicate that Slitrk mutants are in general intracellularly trapped in the *cis*-Golgi and/or ER in non-neuronal cells. To ensure that these Slitrk mutations had a similar effect on the transport of Slitrks out of the ER in neurons, we transfected cultured hippocampal neurons with WT or mutant Slitrks and analyzed them by immunocytochemistry (**Figure 6**). HA-tagged WT Slitrk1 and Slitrk2 were efficiently transported into dendrites, whereas HA-Slitrk1[N400I], HA-Slitrk1[T418S] or HA-Slitrk2[V89M] were either completely retained in the cell body of transfected neurons or displayed markedly impaired trafficking into dendrites (**Figures 6A,B**), as judged by the comparable immunofluorescence signals in the soma of transfected neurons (**Figure 6C**). Notably, other Slitrk2 mutants were targeted to dendrites similarly to Slitrk2 WT (**Figures 6B,C**). These observations suggest that the surface transport deficiency observed in heterologous cells (except for HA-Slitrk2[V89M]) is similarly recapitulated in cultured hippocampal neurons. We were unable to examine the distribution of WT or mutant forms of HA-Slitrk4 because the expression levels of these constructs were too low to be evaluated in this type of analysis.

### Neuronal Phenotypes of Disease-Associated Slitrk Missense Mutants

**FIGURE 7:**
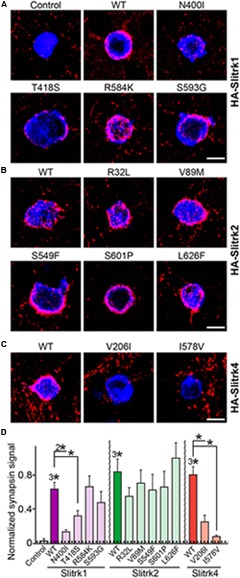
**Impaired synaptogenic activity of a subset of Slitrk mutants in cultured hippocampal neurons. (A-C)** Representative images of the heterologous synapse-formation activities of WT Slitrk and the indicated point mutants in cultured hippocampal neurons. Neurons were cocultured with HEK293T cells transfected with WT or the indicated mutant forms of HA-Slitrk1 **(A)**, HA-Slitrk2 **(B)**, or HA-Slitrk4 **(C)** at DIV10. Neurons at DIV12 were then immunostained with antibodies against EGFP or HA (blue) and synapsin (red). Scale bar, 10 μm (applies to all images). **(D)** Synapse-formation activity in panels **(A–C)** was quantified by measuring the ratio of synapsin staining intensity (red) to HA/EGFP intensity (blue). All data are shown as means ± SEMs (^∗^*p* < 0.05; ^2∗^*p* < 0.01; ^3∗^*p* < 0.001; ANOVA with *post hoc* Tukey’s test; *p*-value of WT Slitrk1 vs. Slitrk1[N400I] = 0.00176; *p*-value of WT Slitrk1 vs. Slitrk1[T418S] = 0.0497; *p*-value of WT Slitrk1 vs. Slitrk1[R584K] = 0.959; *p*-value of WT Slitrk1 vs. Slitrk1[S593G] = 0.434; *p*-value of WT Slitrk2 vs. Slitrk2[R32L] = 0.818; *p*-value of WT Slitrk2 vs. Slitrk2[V89M] = 0.957; *p*-value of WT Slitrk2 vs. Slitrk2[S549F] = 0.956; *p*-value of WT Slitrk2 vs. Slitrk2[S601P] = 0.984; *p*-value of WT Slitrk2 vs. Slitrk2[L626F] = 0.987; *p*-value of WT Slitrk4 vs. Slitrk4[V206I] = 0.00062; and *p*-value of WT Slitrk4 vs. Slitrk4[I578V] = 1.5112E-7). ‘*n*’ denotes the number of HEK293T cells as follows: Control, *n* = 14; WT Slitrk1, *n* = 14; Slitrk1[N400I], *n* = 12; Slitrk1[T418S], *n* = 14; Slitrk1[R584K], *n* = 14; Slitrk1[S593G], *n* = 21; WT Slitrk2, *n* = 12; Slitrk2[R32L], *n* = 10; Slitrk2[V89M], *n* = 10; Slitrk2[S549F], *n* = 15; Slitrk2[S601P], *n* = 15; Slitrk2[L626F], *n* = 15; WT Slitrk4, *n* = 17; Slitrk4[V206I], *n* = 13; and Slitrk4[I578V], *n* = 15.

**FIGURE 8:**
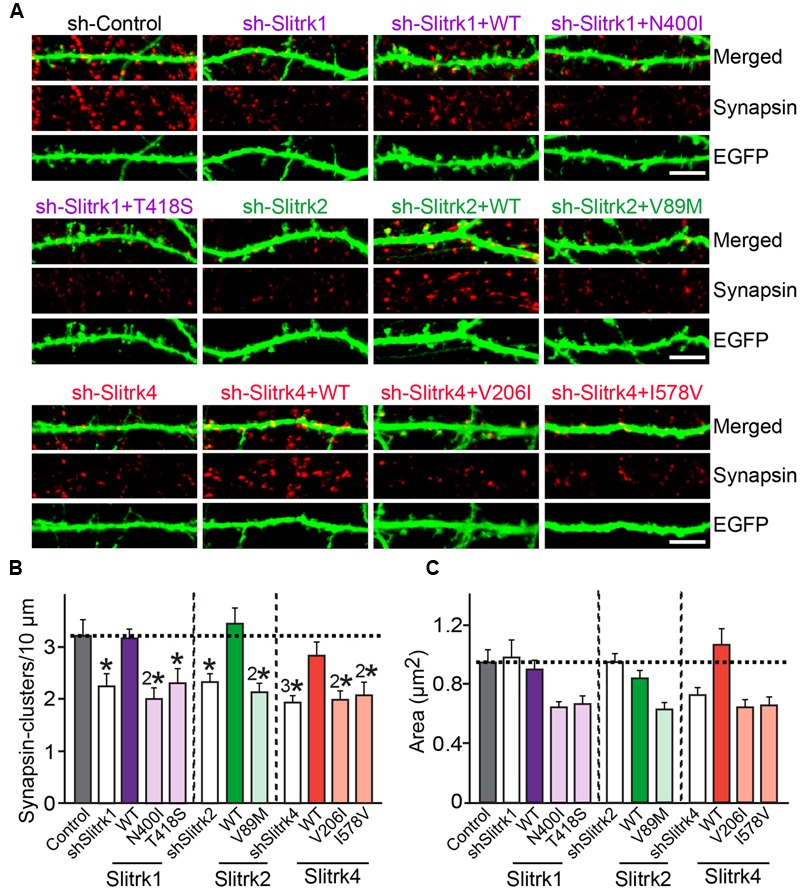
**Impaired regulation of synapse density by a subset of Slitrk mutants in cultured hippocampal neurons. (A)** Cultured hippocampal neurons were transfected with a lentiviral vector expressing sh-Control, sh-Slitrk1, sh-Slitrk2 or sh-Slitrk4, or coexpressing the indicated sh-Slitrk vector and corresponding shRNA-resistant Slitrk vectors at DIV8 and analyzed at DIV14 by double-immunofluorescence staining with antibodies to EGFP (green) and synapsin (red). Scale bar, 5 μm (applies to all images). **(B,C)** Summary data showing the effects of Slitrk molecular replacement in neurons on synapsin puncta density **(B)** and synapsin puncta size **(C)**. More than three dendrites per transfected neuron were analyzed and group-averaged. All data shown are means ± SEMs (^∗^*p* < 0.05; ^2∗^*p* < 0.01; ^3∗^*p* < 0.001; ANOVA with *post hoc* Tukey’s test; *p*-value of sh-Control vs. sh-Slitrk1 = 0.029; *p*-value of sh-Control vs. sh-Slitrk1+WT Slitrk1 = 0.99; *p*-value of sh-Control vs. sh-Slitrk1+Slitrk1[N400I] = 0.004; *p*-value of sh-Control vs. sh-Slitrk1+Slitrk1[T418S] = 0.049; *p*-value of sh-Control vs. sh-Slitrk2 = 0.039; *p*-value of sh-Control vs. sh-Slitrk2+WT Slitrk2 = 0.99; *p*-value of sh-Control vs. sh-Slitrk2+Slitrk2[V89M] = 0.0093; *p*-value of sh-Control vs. sh-Slitrk4 = 0.00075; *p*-value of sh-Control vs. sh-Slitrk4+WT Slitrk4 = 0.877; *p*-value of sh-Control vs. sh-Slitrk4+Slitrk4[V206I] = 0.003; and *p*-value of sh-Control vs. sh-Slitrk4+Slitrk4[I578V] = 0.008). ‘*n*’ denotes the number of neurons as follows: sh-Control, *n* = 16, sh-Slitrk1, *n* = 14, sh-Slitrk1+WT Slitrk1, *n* = 14; sh-Slitrk1+Slitrk1[N400I], *n* = 14; sh-Slitrk1+Slitrk1[T418S], *n* = 14; sh-Slitrk2, *n* = 13; sh-Slitrk2+WT Slitrk2, *n* = 13; sh-Slitrk2+Slitrk2[V89M], *n* = 13; sh-Slitrk4, *n* = 17; sh-Slitrk4+WT Slitrk4, *n* = 15; sh-Slitrk4+Slitrk4[V206I], *n* = 15; and sh-Slitrk4+Slitrk4[I578V], *n* = 15.

**FIGURE 9:**
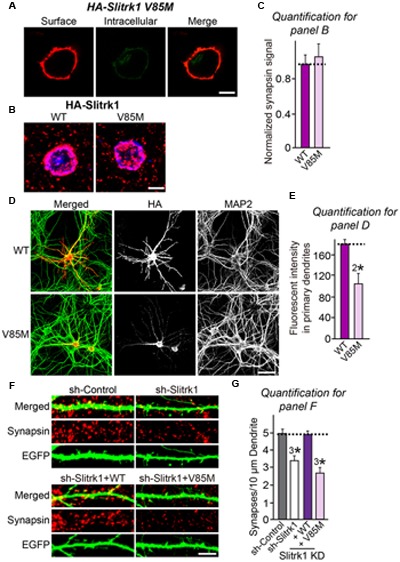
**Characterization of the Slitrk1[V85M] mutant in heterologous synapse-formation assays and transfected neurons. (A)** Surface expression analysis of HEK293T cells expressing HA-tagged Slitrk1[V85M]. Transfected cells were immunostained with mouse anti-HA antibodies (red) and detected with Cy3-conjugated anti-mouse secondary antibodies under non-permeabilized conditions, followed by permeabilization of cells. Cells were then stained first with rabbit anti-HA antibodies (green) and then with FITC-conjugated anti-rabbit secondary antibodies. Scale bar, 10 μm (applies to all images). **(B,C)** Representative images **(B)** and summary graph **(C)** of heterologous synapse-formation activities of WT Slitrk1 and Slitrk1[V85M]. Neurons were stained with antibodies against HA (blue; pseudo-colored) and synapsin (red). Scale bar, 10 μm (applies to all images). ‘*n*’ denotes the number of HEK293T cells analyzed: WT Slitrk1, *n* = 13; and Slitrk1[V85M], *n* = 12. **(D)** Fluorescence images of hippocampal neurons transfected with WT HA-Slitrk1 or HA-Slitrk1[V85M], at DIV8. After 48 h (DIV10), the transfected neurons were double-immunostained for antibodies against the somatodendritic marker MAP2 (green) and HA (red). Note that Slitrk1[V85M] exhibits decreased dendritic targeting. Scale bar, 50 μm (applies to all images). **(E)** Dendritic targeting of WT HA-Slitrk1 or HA-Slitrk1[V85M] in hippocampal neurons was quantified by measuring fluorescent intensity (HA) in primary dendrites. All data are shown as means ± SEM (^2∗^*p* < 0.01; Student’s t-test; *p*-value = 0.00215). ‘*n*’ denotes the number of neurons as follows: WT Slitrk1, *n* = 15 and Slitrk1[V85M], *n* = 13. **(F,G)** Representative images **(F)** and summary graph **(G)** of neuron transfection assays with a lentiviral vector expressing sh-Control, sh-Slitrk1, or coexpressing sh-Slitrk1 and the indicated shRNA-resistant Slitrk1 vectors at DIV8 and analyzed at DIV14 by double-immunofluorescence staining with antibodies to EGFP (green) and synapsin (red). Scale bar, 5 μm (applies to all images). All data are shown as means ± SEMs (^3∗^*p* < 0.001; ANOVA with *post hoc* Tukey’s test; *p*-value of sh-Control vs. sh-Slitrk1 = 0.000411; *p*-value of sh-Control vs. sh-Slitrk1+WT Slitrk1 = 0.991; and *p*-value of sh-Control vs sh-Slitrk1+Slitrk1[V85M] = 2.74E^-7^). ‘*n*’ denotes the number of neurons quantified as follows: sh-Control, *n* = 15, sh-Slitrk1, *n* = 15, sh-Slitrk1+WT Slitrk1, *n* = 15; and sh-Slitrk1+Slitrk1[V85M], *n* = 17.

Slitrks were previously shown to trigger presynaptic differentiation when expressed in heterologous cells and cocultured in contact with axons (Takahashi et al., 2012; Yim et al., 2013). To test whether the surface transport-deficient Slitrk mutants showed differences in presynaptic differentiation-inducing behavior compared with WT Slitrks, we performed heterologous synapse-formation assays with HEK293T cells expressing WT or mutant Slitrks (**Figure 7**). We found that, whereas WT Slitrk1, Slitrk2, and Slitrk4 robustly recruited synapsin clustering into the corresponding transfected HEK293T cells, Slitrk1[N400I] and Slitrk1[T418S] as well as Slitrk4[V206I] and Slitrk4[I578V] mutants were functionally inactive, as expected by their lack of surface transport (**Figures 7A,C**; see quantification in **Figure 7D**). None of the tested Slitrk2 mutants exhibited altered synapse-inducing activity (**Figures 7B,D**). To determine whether the surface transport-impairing Slitrk mutations also compromised the ability of WT Slitrk proteins to promote synapse formation in cultured neurons, we introduced Slitrk isoform-specific knockdown (KD) vectors into cultured hippocampal neurons at 8 days *in vitro* (DIV8), and stained the transfected neurons with anti-EGFP and anti-synapsin antibodies at DIV14 (**Figure 8**). As previously reported (Yim et al., 2013), single KD of Slitrk1, Slitrk2, or Slitrk4 significantly decreased the linear density of synapsin clusters (**Figures 8A,B**). Expression of short hairpin RNA (shRNA)-resistant forms of WT Slitrk1, Slitrk2, or Slitrk4 completely rescued these deficits in the numbers of synapsin clusters (**Figures 8A,B**). Similar to the results of heterologous synapse-formation assays, expression of shRNA-resistant Slitrk1 mutants (N400I or T418S) or Slitrk4 mutants (V206I or I578V) failed to reverse the reduction in the density of synapsin clusters induced by knockdown of Slitrk1 or Slitrk4 (**Figures 8A,B**). Strikingly, expression of the Slitrk2[V89M] mutant, which showed total expression levels, surface transport, and synapse-inducing properties comparable to those of WT Slitrk2, did not rescue the Slitrk2 KD-induced deficit in the numbers of synapsin clusters, likely due to impaired dendritic targeting in neurons (**Figures 8A,B** and **3**). Since the valine residue at position 89 in human Slitrk2 is also found Slitrk1, -3 and -4 (but not Slitrk5 and- 6; **Figure 1B**), we introduced a similar point mutation into human Slitrk1 (Slitrk1[V85M]) and examined whether this artificially generated Slitrk1 mutant behaves similarly to Slitrk2[V89M] (**Figure 9**). We found that Slitrk1[V85M] showed surface expression levels and synaptogenic activity comparable to those of WT Slitrk1 (**Figures 9A–C**), but exhibited decreased dendritic targeting in hippocampal neurons (**Figures 9D,E**). Moreover, Slitrk1[V85M] showed impaired synapse-promoting activity when expressed in Slitrk1-deficient neurons (**Figures 9F,G**). These data suggest that different Slitrk variants found in patients with neuropsychiatric disorders may manifest distinct cellular readouts, compounding the enormous complexity at both cellular and behavioral levels.

## Discussion

The ‘synaptopathy’ hypothesis has been dominant in the neuroscience field since the term was coined, aided in part by the rapid advent of high-resolution human genetic sequencing technologies (Brose et al., 2010). Various classes of synaptic genes have been associated with a range of neuropsychiatric and neurodevelopmental disorders, particularly in the case of ASDs and schizophrenia, although most copy number variants are rarely found in patients with these disorders (Glessner et al., 2012; Malhotra and Sebat, 2012). Surprisingly, similar genetic pathways have often been found to be linked to phenotypically distinct outcomes, confounding the interpretation of genetic approaches (Marshall and Scherer, 2012). Specifically, synaptic adhesion molecules and their associated scaffold proteins, such as neurexin-1α, neuroligin-4 and Shank3, are among the few synaptic genes that are frequently identified as causative factors for ASDs and schizophrenia (Sudhof, 2008; Won et al., 2013; de la Torre-Ubieta et al., 2016). However, how these disease-susceptible genes cause the associated disorders has only recently begun to be understood.

In the present study, we employed a series of functional approaches to ask whether Slitrk mutations identified in schizophrenia, trichotillomania or OCDs alter the biochemical, cellular and synaptic processes that are mediated by WT Slitrk proteins. We found that a subset of Slitrk1, Slitrk2, and Slitrk4 missense mutants exhibit common and distinct phenotypes in a variety of assays, results that often differed from those predicted by algorithm-based *in silico* analyses (**Table 1**). Indeed, many of these mutations did not induce any prominent alterations in the properties of Slitrk proteins, although it is possible that the functional assays employed in the current study were unable to capture all aspects of Slitrk function. Thus, our data underscore the importance of experimentally testing whether candidate mutants identified based on human genetics are physiologically significant. In exploring whether candidate human mutations have distinctive phenotypes at molecular and cellular levels, we focused on two Slitrk1 missense mutations (N400I and T418S), a single Slitrk2 mutation (V89M), and two Slitrk4 mutations (V206I and I578V).

Our data suggest that the respective disorder phenotypes observed in patients with Slitrk mutations are at least partly caused by Slitrk dysfunctions, based on the following evidence: (1) most of the mutated residues in Slitrk1, Slitrk2, and Slitrk4 are evolutionarily conserved across various species, although only a few are also found across all six Slitrk family members; (2) most of the substitutions caused retention in intracellular compartments, such as the ER and *cis*-Golgi, with an accompanying loss of normal Slitrk glycosylation patterns; (3) none of the substitutions perturbed ligand-binding properties, judging from previously determined complex structures; and (4) all but one of the substitutions abolished the effects of Slitrks on synapse formation, with Slitrk2[V89M] uniquely exhibiting the ability to trigger presynaptic differentiation. Notably, none of the Slitrk mutations described here abolished binding to LAR-RPTPs *per se*. However, a majority of Slitrk mutations are positioned in the LRR2 domain of Slitrks (**Figure 1**); thus, the possibility that LRR2 domain-mediated molecular interaction(s) might be influenced by Slitrk mutations cannot be excluded at this point. At present, precisely how Slitrks promote distinct types of synapse development in an isoform-dependent manner remains to be elucidated (Yim et al., 2013), but it would be worthwhile investigating the relationship between altered synapse numbers induced by Slitrk missense mutations and the development of associated neuropsychiatric disorders in follow-up studies. Intriguingly, Slitrk family proteins resemble neuroligin family proteins in many ways (Brose, 2013). For example, the action of Slitrk3 at inhibitory synapses is phenomenologically analogous to that of neuroligin-2, whereas the effects of the other Slitrk family members are related to those of neuroligin-1. However, more rigorous analyses should be undertaken to provide a complete understanding of how synaptogenic adhesion molecules operate cooperatively, competitively, or both.

Most Slitrk substitutions represent loss-of-function mutations that perturb the normal folding and glycosylation of Slitrks, seemingly similar to the situation previously described for the R87W substitution in human neuroligin-4 and D1129H substitution in human CNTNAP2 (Zhang et al., 2009; Falivelli et al., 2012). The V89M substitution is somewhat analogous to previous descriptions of the R451C substitution in human neuroligin-3 (Comoletti et al., 2004; De Jaco et al., 2010) in that Slitrk2[V89M] protein retained WT-like presynapse-inducing activity (**Figure 7**). However, this substitution acted as dominant-negative mutation, abolishing the ability of Slitrk2 to promote synapse formation in transfected neurons because of its ability to alter the synaptic properties of neurons (**Figure 8**). Intriguingly, our preliminary analyses indicate a weak correlation between dendritic spine density and synapsin puncta density in Slitrk-deficient neurons (Data not shown). Further investigation using membrane-anchored GFP plasmid to more accurately visualize the dendritic spines would be required to solve the seemingly discrepancy between postsynaptic spine density and presynaptic marker density.

**FIGURE 10:**
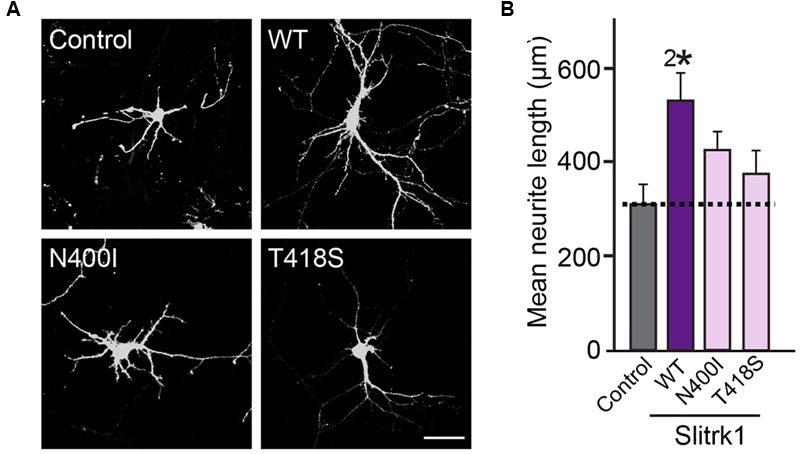
**The effect of the Slitrk1[N400I] and Slitrk1[T418S] mutants on neurite outgrowth in cultured hippocampal neurons. (A)** Representative images of DIV6 hippocampal neurons transfected with WT HA-Slitrk1, HA-Slitrk1[N400I], or HA-Slitrk1[T418S] at DIV3. Scale bar, 50 μm (applies to all images). **(B)** Quantification of total neurite length per hippocampal neuron at DIV6. All data are shown as means ± SEMs (^2∗^*p* < 0.01; a non-parametric Kruskal-Wallis test; *p*-value of Control vs. WT Slitrk1 = 0.003; *p*-value of Control vs. Slitrk1[N400I] = 0.0634; *p*-value of Control vs. Slitrk1[T418S] = 0.300). ‘*n*’ denotes the number of neurons as follows: Control, *n* = 15, WT Slitrk1, *n* = 16, Slitrk1[N400I], *n* = 14; and Slitrk1[T418S], *n* = 14.

Currently, we have no insight into how the V89M substitution causes inactivation of Slitrk2 function, but a similar valine-to-methionine mutation was previously described for BDNF (brain-derived neurotrophic factor) (Bath and Lee, 2006; Chen et al., 2006). The human BDNF[V66M] polymorphism, which is associated with altered dendritic trafficking of BDNF mRNA, changes in hippocampal volume, impaired hippocampal-dependent memory and NMDA-receptor dependent synaptic plasticity, and extinction of conditioned aversive memory, has been implicated in anxiety disorders (Chiaruttini et al., 2009; Yu et al., 2009; Ninan et al., 2010). The pathophysiological mechanism postulated for the effect of this BDNF mutation is that sortilin directly interacts with WT BDNF, but not BDNF[V66M], in secretory granules and controls the pathway that regulates its secretion in neurons (Chen et al., 2005). However, Slitrk2[V89M] does not block the transport of Slitrk2 protein to the cell surface; thus, it is conceivable that other mechanism(s) may operate in this case. Slitrk1[N400I] was previously shown to be unable to stimulate neurite outgrowth (Ozomaro et al., 2013), and the current study also found that its glycosylation, surface transport, and synaptogenic activities were impaired (**Figures 3–8**). In contrast, Slitrk1[T418S] was not previously examined, because it was frequently detected in individuals without OCDs and thus was considered to be functionally tolerated in the general population (Ozomaro et al., 2013). However, our data clearly suggested that the T418S mutation also abolishes the surface transport of Slitrk1 proteins, possibly by impairing glycosylation patterns, similar to the biochemical and cellular phenotypes of the N400I mutation. More intriguingly, the Slitrk1[T418S] mutation also compromised the enhanced neurite outgrowth activity shown by Slitrk1 WT (**Figure 10**).

Apart from the unique action of the Slitrk2 V89M mutation, the other Slitrk1, Slitrk2, and Slitrk4 mutations described in the current study have features in common that are typical of loss-of-function mutations, consistent with the phenotypes of Slitrk KD in cultured hippocampal neurons (Yim et al., 2013). Remarkably, Slitrk1-knockout (KO) mice exhibit elevated anxiety-like behaviors that are attributable to increased norepinephrine levels, and administration of the centrally acting adrenergic agonist clonidine has been shown to normalize these behaviors (Katayama et al., 2010). However, the core symptoms of Tourette syndrome and trichotillomania, such as self-grooming, were not reported in Slitrk1-KO mice (Katayama et al., 2010). In the case of neuroligin-3, the R451C mutation exhibits distinct gain-of-function synaptic phenotypes compared with neuroligin-3-KO mice, but both neuroligin-3 R451C knock-in (KI) and KO also impair tonic endocannabinoid signaling in specific interneuron-type synapses (Foldy et al., 2013). Thus, a worthwhile task would be to compare the behavioral, physiological, and circuit phenotypes of Slitrk-KI mice harboring some of the point mutations with altered Slitrk functions described in the current study with those obtained from Slitrk-KO mice. These approaches hold the potential of unveiling more detailed pathophysiological mechanisms underlying Slitrk-associated neuropsychiatric disorders.

## Author Contributions

JK and JWU conceived and supervised the project; HK, KAH, SYW, and JWU performed the experiments; HK, KAH, HMK, YHL, JK, and JWU analyzed the data; JK and JWU wrote the paper. All authors were involved in drafting the paper and provided final approval of the version to be submitted.

## Conflict of Interest Statement

The authors declare that the research was conducted in the absence of any commercial or financial relationships that could be construed as a potential conflict of interest.

## Footnotes

1 http://genetics.bwh.harvard.edu/pph2/

2 http://provean.jcvi.org/index.php

3 http://mutationassessor.org/r3/

4 http://www.pantherdb.org/tools/
